# Endoscopic Submucosal Dissection in the Upper Gastrointestinal Tract and the Need for Rescue Surgery—A Multicenter Analysis

**DOI:** 10.3390/jcm12216940

**Published:** 2023-11-06

**Authors:** Philipp Pimingstorfer, Matthias Biebl, Matus Gregus, Franz Kurz, Rainer Schoefl, Andreas Shamiyeh, Georg O. Spaun, Alexander Ziachehabi, Reinhold Fuegger

**Affiliations:** 1 Kepler Universitätsklinikum, 4020 Linz, Austria; 2 Department of Surgery, Ordensklinikum Linz Barmherzige Schwestern, 4010 Linz, Austria; 3 Department of Gastroenterology, Ordensklinikum Linz Barmherzige Schwestern, 4010 Linz, Austriarainer.schoefl@ordensklinikum.at (R.S.)

**Keywords:** ESD outcome, ESD upper GI, rescue surgery

## Abstract

Endoscopic submucosal dissection (ESD) has become the standard treatment for early malignant lesions in the upper gastrointestinal (GI) tract. Its clinical results have been reported to be as good as surgery. The outcomes of rescue surgery after non-curative ESD have been reported to be as good as first-line surgery. The aim of this study was to evaluate the outcomes of ESD in the upper GI tract and the outcomes of rescue surgery after non-curative ESD performed in Linz, Austria, between 2009 and January 2023. A total of 193 ESDs were included and divided into 104 esophageal ESD and 89 gastric ESD procedures. The criteria for curative ESD were in line with established guidelines’ recommendations. For esophageal lesions, the mean lesion size was 40.3 mm and the rate of curative ESD was 56.7%. In the non-curative ESD, the rate of technical failure as the reason for non-curative ESD was 13.3% and the oncological failure rate was 86.7%. Only 48.7% of indicated rescue surgeries were performed. The main reason for not performing surgery was interdisciplinary consensus due to comorbidity. Perioperative complications Dindo–Clavien ≥ 3 occurred in 22.2% of cases with an in-hospital mortality rate of 0. In gastric lesions, the mean size was 39 mm and the rate of curative ESD was 69.7%. The rate of technical failure as a reason for non-curative ESD was 25.9% and the oncological failure rate was 74.1% for non-curative ESD. Rescue surgery was performed in 48.2% of indicated cases. The perioperative rate for major complications was 0. The outcome of ESD in the upper GI tract is in line with the published literature, and non-curative ESD does not worsen surgical outcomes. The available follow-up data are in line with the international published literature, showing a low rate of residual malignancy in surgical resection specimens. Therefore, the indication of rescue surgery for oncological failure remains challenging. Furthermore, the learning curve of ESD has shown a trend towards improving outcomes over time.

## 1. Introduction

Therapeutic strategies for early cancer of the upper gastrointestinal (GI) tract are evolving over time. With recent improvements in the detection of early malignant lesions with advances in technology, endoscopic resection, such as endoscopic mucosal resection (EMR) and endoscopic submucosal dissection (ESD), has become the standard treatment in early malignant lesions in the upper GI tract. Its oncological results have been reported to be as good as surgery, with a favourable safety profile when comparing ESD to surgery [1,2].

The risk of disease recurrence is strongly associated with histopathological risk factors in resection specimens, such as tumor differentiation, the invasion depth in the submucosal layer, the infiltration of vessels, or lymphovascular infiltration [3,4,5,6,7,8,9]. Those criteria are associated with the risk of lymph node metastasis. Guidelines defining the indication for ESD follow the main principle that the risk of recurrence by lymph node metastases must be lower than the risk of death following major surgery. Endoscopic resection of early malignant lesions has become the standard of treatment in most Western parts of the world and in East Asia. 

However, especially in the early phase of ESD, the role of surgery as a rescue procedure for inappropriate oncological ESD and the complications of ESD, like perforation, was largely unknown.

In this descriptive retrospective analysis, we aim to evaluate the outcome of ESD for early cancer of the upper GI tract in the federal state of Upper Austria and the indication and results of rescue surgery. Furthermore, we investigated the indications of rescue surgery regarding the previous technical failure or oncological failure of ESD and we investigated the perioperative outcomes of rescue surgery. 

To our knowledge, no Austrian data on ESD for early malignant lesions or rescue surgery after ESD have been published so far.

## 2. Methods

We performed a multicenter retrospective descriptive analysis of all ESD procedures performed in esophageal and gastric lesions between 2009 and January 2023. The participating hospitals were Ordensklinikum Linz—Barmherzige Schwestern (Linz, Austria), Ordensklinikum Linz—Elisabethinen (Linz, Austria), and the Kepler University Clinic (Linz, Austria). 

To identify all ESD procedures, we searched all endoscopic resections in the upper gastrointestinal tract in each hospital’s database for billing data to the relevant social insurance provider. After the exclusion of polypectomies and EMR, a combined database of all ESD procedures was created using Microsoft Excel 2016. For data protection, we used pseudonymization in the database. For clinical classification of the lesions, we noted the lesion size and localization. The technical success of ESD was defined as en-bloc resection with tumor-free specimen margins without perforation. We checked the histopathologic results and distinguished between malignant and non-malignant lesions. We defined gastric adenomas with low- or high-grade dysplasia as well as esophageal lesions with low- or high-grade dysplasia as non-malignant. 

For malignant lesions, we defined curative resection or non-curative resection as per established criteria from international guidelines. The criteria for curative resection of early malignant lesions in the upper gastrointestinal tract [3,4,5,6,7,8,9,10] differ between the esophagus and stomach, the histopathological type of the tumor, and technical standards.

The first criterion is the en-bloc resection of the lesion with malignancy-negative horizontal and lateral margins of the resection specimen. Regarding the risk of lymph node metastasis, the risk differs between organs, and an oncological curative resection is defined for:

- Squamous cell carcinoma in the esophagus: pT1b (sm1) with an invasion depth < 200 µm in the submucosal layer without risk factors (L0 and V0) and tumor differentiation of G1 or G2.
- Barrett’s carcinoma or carcinomas of the esophagogastric junction: pT1b (sm1) with an infiltration depth up to 500 µm in the submucosal layer, L0, V0, and tumor differentiation of G1 or G2.
- Gastric carcinoma: Differentiated non-ulcerated tumor < 2 cm diameter and L0 and V0 with up to a maximum of one of the expanded criteria: pT1b (sm1) with an infiltration depth up to 500 µm in the submucosal layer, a non-ulcerated lesion independent of size, a differentiated ulcerated lesion < 3 cm diameter, or an undifferentiated lesion < 2 cm diameter.

Before the ESD procedure, each patient underwent a second-look endoscopy carried out by an expert endoscopist to check the indication and feasibility of ESD according to the optical criteria. In potentially advanced lesions, a staging endoscopic ultrasound was performed. If the lesion was classified as non-eligible for ESD (e.g., ulceration, signs of deep submucosal infiltration via a vascular pattern, or positive lymph nodes in the endoscopic ultrasound), the ESD was not performed. 

If a resection was deemed non-curative when following the above-mentioned criteria, we discussed the individual patient at the interdisciplinary tumor conference. When the recommendation was for rescue surgery, patient charts were analyzed for the type of surgery, definite histology, and perioperative complications. We documented the reason given for patients for whom rescue surgery was indicated and recommended but not performed.

Available follow-up data were noted in the database. As all participating hospitals are tertiary care centers, several patients were referred for ESD. Regarding these referred patients, rescue surgery after the oncological failure of ESD was performed in other hospitals in Austria individually. The outcome data of these patients who had surgery in other hospitals were requested and included in our analysis. We did not request outcome data of patients who had undergone follow-up after ESD without rescue surgery in other hospitals. The complications of rescue surgery were graded according to the Dindo—Clavien classification [11].

Statistical analysis to calculate ESD outcomes and perioperative outcome ratios was performed using Microsoft Excel 2016. The first ESDs in Austria were performed around 2009. Assuming a learning curve of five years to gain expertise in the field, we divided the cohort in the second step into two groups, from 2009–2014 and 2015–2023, to check for the learning curve of procedures by the rate of successful ESD, curative resections, and periinterventional complications.

As this is a retrospective, descriptive, non-interventional study, IRP approval was not necessary. 

## 3. Results

A total of 193 ESD procedures for early esophageal or gastric lesions were performed during the study period. The mean age of the patients was 71.9 (12.6), of whom 75.1% were male. The procedures were divided into 104 esophageal ESD and 89 gastric ESD procedures. 

The esophageal lesions measured a mean of 40.3 mm (max. 115 mm). Approximately 80% of the esophageal lesions were Barrett’s associated lesions in the lower third of the esophagus. Approximately 20% of the lesions (n = 21) were squamous epithelial lesions; the location for the lower third, middle third, and upper third comprised 1, 16, and 4, respectively. Regarding the outcomes of esophageal ESD (Table 1), the en-bloc resection rate was 93.3% and the rate of curative resection of all esophageal lesions was 56.7%.

Peri-interventional complications occurred in 6.7% of patients and were mainly post-procedural bleeding, all of which were managed endoscopically. Three patients developed post-interventional esophageal stricture, which was managed via balloon dilatation. No perforation occurred in the cohort. 

The rate of malignant esophageal lesions was 81.7% and the rate of curative resections in malignant lesions was 47.1%. Dysplastic lesions (high-grade or low-grade dysplasia) were considered as non-malignant. Only two resection specimens of ESD showed a non-malignant non-dysplastic histopathology (squamous cell epithelia).

Of those with non-curative esophageal ESD procedures with en-bloc resection (43.3%), eight patients had histopathological lateral R1 or Rx even though there was macroscopic complete resection. All of these lesions were Barrett’s associated lesions. One of those had rescue surgery, without a residual tumor left. One patient subsequently had another ESD, without any evidence of residual malignancy or dysplasia. The other six patients had surveillance endoscopies every three months in the first year. The available follow-up data comprise a mean observation period of 11 months. One of the six patient had malignant recurrence but could be managed curatively by a subsequent ESD procedure.

Surgery was indicated in 35.6% (n = 37) of patients after esophageal ESD. The indication for surgery was technical failure of ESD in 6 cases and oncological failure in 31 cases. Technical failure was a resection that was not en-bloc, perforation, or macroscopic signs of residual malignancy. Oncological failure was defined by the criteria described above. The reasons for oncological failure were (in regard for multiple causes) poor tumor differentiation (G3-4) (n = 22), lymphovascular infiltration (n = 6), deep submucosal infiltration (submucosal invasion >200 µm for squamous lesions and >500 µm for Barrett’s associated lesions) (n = 9), and R1 in the deep resection margin (n = 8). 

Overall, rescue surgery following esophageal ESD was only performed in 17.3% (n = 18), thus in approximately half of the indicated cases. Regarding the outcome of rescue surgery (Table 2), 12 of those 18 patients had no residual malignancy and negative lymph nodes. Surgical complications graded Dindo–Clavien ≥ 3 occurred in four patients. Three patients suffered from anastomotic leak, and one suffered from necrosis of the interponated stomach. In-hospital mortality did not occur. 

The main reasons for not performing rescue surgery were interdisciplinary consensus at the tumor conference due to comorbidity (n = 16) and individual patients’ decision against surgery (n = 3).

In the gastric lesions, the mean lesion size was 39 mm (max 85 mm) and 28% of the gastric lesions were located in the cardia or fundus area. Regarding the outcome of gastric ESD (Table 3), the en-bloc and curative resection rates were 92.1% and 69.7%, respectively. Complications occurred in 12.4% of cases; five of those were delayed bleeding within one week, which could be managed via endoscopy. There were four perforations observed in gastric ESD, three of which required rescue surgery and one which could be managed successfully via endoscopy. One patient had free peritoneal air after ESD, but no signs of peritonitis or perforation and was managed successfully conservatively.

The rate of malignancy in the resection specimens for gastric lesions was 66.3% (n = 59); six of those patients had a neuroendocrine tumor (NET) and two had gastrointestinal stroma tumors (GIST). The two GIST were small (<2 cm) and had a low-risk histopathological assessment (<5 mitosis/HPF) in a previous EUS-guided biopsy and were therefore treated by means of ESD. Two of those patients with NET were non-curative by ESD and required additional surgery because of their intra-procedural cT2 staging. Dysplastic lesions (high-grade or low-grade dysplasia) were considered as non-malignant. 

Rescue surgery was indicated in 30.3% (n = 27) of patients. The indication for surgery was the technical failure of ESD in seven cases, of which four cases were perforations, two cases were intraprocedural cT2 staging, and one case was severe submucosal fibrosis with macroscopic incomplete resection. The oncological failure (as defined above) of ESD occurred in 20 patients. The reasons for oncological failure were (multiple causes possible) poor tumor differentiation (G3-4) (n = 5), lymphovascular invasion (n = 5), deep submucosal invasion (>500 µm) (n = 13), and a horizontal R1 margin (n = 4). 

Rescue surgery was performed in 13 (48.1%) patients of the 27 with an indication. The main reason for not performing surgery was interdisciplinary consensus due to comorbidity (n = 13), and one patient refused surgery after giving informed consent. Follow-up data of those without rescue surgery were only available for five patients with a mean follow up time of 6 months, without any evidence of tumor recurrence. The main reason for not having more follow-up data available is, that as reference hospitals, many patients underwent follow-up in other hospitals.

Regarding the outcome of rescue surgery after non-curative gastric ESD (Table 2), residual malignancy was present in four patients. Each of those had lymph node metastasis. No major complications (Dindo–Clavien ≥ 3) or in-hospital mortality were observed. Follow-up data on surgery and histopathology were available for 10 of the 13 patients.

At the beginning of the observational period, ESD had recently been implemented in Austria. To assess the learning curve of the technique of ESD, we assumed a duration of five years to gain expertise in the field. Therefore, we divided the cohort into two groups, 2009–2014 (cohort A) and 2015–2023 (cohort B). The number of ESD procedures for cohort A was 54, and for cohort B, it was 139. The rates of successful ESD, curative ESD, and complications were 87% vs. 94%, 61% vs. 63%, and 13% vs. 6%, respectively. Using Chi-square testing, the *p*-values were 0.14, 0.78 and 0.09, respectively. The cohort was not powered to show statistical significance, but we were able to show a trend towards better outcomes in more recent years.

## 4. Discussion

Today, ESD of early malignant and premalignant lesions of the esophagus and stomach is an established procedure. Its oncologic results are comparable to major surgery, but ESD is clearly superior regarding perioperative complications, interventional trauma, and post-procedure organ function.

For mucosal esophageal squamous cell carcinomas with invasion until lamina propria (m2), the rates of curative ESD vary around 73.5% and the 5-year disease free survival rate is 95.2% [12]. In comparison to surgery, there are fewer severe complications in the ESD group in the published literature [13]. The efficacy of ESD in Barrett’s associated neoplasia is well described. A meta-analysis comparing the outcomes of ESD between Eastern and Western countries reveals better results for the East. Primarily, this may be caused by the greater experience Eastern centers have in dealing with ESD, documented by markedly higher patient numbers and the longer time period of practicing ESD [14]. 

Large multicenter studies from North America and Germany report en-bloc resection rates for ESD of the esophagus of 91.5% and 92.4% and curative resection rates of 78.3% and 72.3%, respectively [15,16]. Considering the inclusion of the learning curve in our cohort, the outcome rates compare well with the international results.

Analyzing the long-term survival of patients with T1 superficial esophageal carcinoma treated by ESD or radical surgery, OS and PFS were significantly longer in the ESD group [17]. A multicentric cohort study including 9054 patients with early gastric cancer reported equal survival for ESD compared to surgery for 5-year OS [18]. Although randomized controlled trials comparing ESD and radical surgery, especially minimally invasive, robotic-assisted surgery, are lacking, ESD provides comparable oncologic results for comparable tumor stages with lower perioperative morbidity and mortality.

The role of surgery in the context of ESD has been discussed from the beginning. Especially in Western countries, there had been concerns regarding the complications of ESD and the need for rescue surgery to handle them. In their meta-analysis, Daoud et al. reported perforation rates requiring surgery of 0.01% and 0.53% for Eastern and Western patients, respectively [14]. Yang et al. published a meta-analysis [19] with a curative resection rate of 64.9%, a perforation rate of 1.5%, and a bleeding rate of 1.7%. Bleeding and perforation are the most common complications observed and are treated endoscopically in the vast majority today. In our series, three patients with gastric ESD were in need of surgery due to perforation. Overall, our rate of rescue surgery due to periinterventional complications was 1.6% (3/193). In accordance with published series, our experience underlines that surgery for complications of ESD is rarely prompted.

However, surgery is of substantial importance in the case of non-curative ESD. Several meta-analyses report short- and long-term outcomes after ESD and the indication for rescue surgery after complications or non-curative ESD. The incidence of non-curative ESD in gastric lesions has been reported to vary between 11.9% and 18.5% [20,21,22,23,24]. Following oncologic principles, all patients with non-curative endoscopic resections should be offered radical surgery for tumor clearance. However, this topic needs more accurate consideration. In our cohort, only 48% of indicated rescue surgeries were performed. The outcomes of rescue surgery after non-curative esophageal ESD are not inferior to first-line surgery. Dermine et al. published a retrospective analysis [25] of rescue surgery after unsuccessful esophageal ESD. Approximately 43% had a major complication (e.g., anastomotic leak), with a 30-day mortality of 3% and a 90-day mortality of 6.7%. Othman MO et al. [26] reported that 10 out of 21 patients with non-curative esophageal ESD subsequently underwent radical surgery. In total, 2 of the 10 patients without surgical resection developed recurrence within a follow-up period of 22.5 months. In our cohort, major complications after esophageal surgery occurred in four patients (22.2% of esophageal surgery) without complication-related mortality. For rescue surgery after non-curative gastric ESD, we did not observe major morbidity or in-hospital mortality.

Summarizing patients with non-curative ESD in our series, about half of them actually had major surgery. The main reasons for not performing rescue surgery were interdisciplinary consensus due to comorbidity and patients refusing surgery after informed consent had been given in some cases. For gastric lesions after non-curative ESD for oncological reasons, in patients who did not undergo surgery, we were able to show follow up data for five patients over 6 months, without recurrence. Two of those patients had non-curative ESD due to lymphovascular infiltration, in two patients, this was because of deep submucosal invasion, and in one patient, this was because of Rx in the basal margin. None of these patients had malignant recurrence over six months. In the published literature, after non-curative ESD for gastric lesions, 5-year survival rates between 72% and 84% have been reported without additional surgery [20,27]. 

Eight patients who had a histopathological R1 or Rx situation in the lateral margin of the resection specimen in esophageal ESD for Barrett’s neoplasia were considered as non-curative, but without a strict indication for surgery. After a tumor board decision was made, two of those had subsequent resection (one ESD and one surgery), each without residual malignancy left. The other six patients had follow-up. The available follow-up data cover 11 months in terms of the mean; only one patient had recurrence of superficial adenocarcinoma and could be managed via another ESD procedure, curatively. These results are in line with the reported results in a cohort analysis of van Munster et al. and justify follow-up in this situation [28].

Another item of concern is the percentage of patients with residual tumors after an ESD which are judged to be non-curative. In our cohort, two patients with non-curative esophageal ESD, one undergoing radical esophagectomy and the other having to redo the ESD procedure, had no residual tumor diagnosed in the subsequent resection specimen. Santos-Antunes et al. [29] reported that a residual tumor was diagnosed in only 13% of patients with non-curative ESD. 

The indication for radical oncologic surgery may be relativized by comorbidities and advanced age. In a retrospective comparison of patients with early gastric cancer older than 75 years with relative indication for ESD, OS was superior for those undergoing surgery, but DFS was not different. Thus, optimizing short-term outcomes at the expense of impaired long-term results may be an arguable option in high-risk patients [30]. The indication for rescue surgery with oncologic indication remains challenging and depends on individual patient characteristics like comorbidities, life-expectancy, and the intentions of the patient.

## 5. Conclusions

The outcomes of ESD for early malignant lesions in the esophagus and stomach in Upper Austria are in line with the international published literature. Rescue surgery due to complications of ESD is prompted rarely. After non-curative ESD, up to 50% of rescue surgeries are not performed due to comorbidity or the patient’s refusal to undergo surgery. In this subpopulation, the indication for rescue surgery for oncological reasons remains a challenging decision due to a low rate of residual malignancy and the OS of elderly patients. Non-curative ESD previous to surgery does not worsen surgical outcomes. Regarding the learning curve of ESD, we were able to show a trend towards better outcomes of ESD with increasing expertise.

## Figures and Tables

**Table 1:** Outcomes of esophageal ESD.

Esophageal ESD	N			%
			104	100
curative			59	56.73
non-curative			45	43.27
	Technical failure		6	13.33
		Perforation	0	
		Piecemeal	4	8.89
		Deep infiltration	2	4.44
		Fibrosis	0	
	Oncological failure (muliple causes)		39	86.67
		G 3-4	22	48.89
		L1	6	13.33
		V1	0	
		>sm1	9	20
		R1 lat	8	17.78
		R1 basal	8	17.78

Abbreviation: ESD—endoscopic submucosal dissection.

**Table 2:** Perioperative outcomes of rescue surgery after ESD.

			Esophageal ESD		Gastric ESD	
			n	%	n	%
surgery indicated			37	35.58	27	30.34
	Lateral R1		8	7.69		
surgery performed			18	48.65	13	48.15
	Outcome					
		Residual tumor	6	33.33	5	38.46
	Complication Dindo–Clavien ≥ 3		4	22.22	0	
		Anastomotic leak	3	16.67		
		Stomach interponate necrosis	1	5.56		
		In-hospital mortality	0		0	

**Table 3:** Outcome of gastric ESD.

Gastric ESD			N	%
			89	
curative			62	69.66
non-curative			27	30.34
	Technical failure		7	25.93
		Perforation	4	14.81
		Piecemeal	2	7.41
		Deep infiltration	0	
		Fibrosis	1	3.70
	Oncological failure		20	74.07
		G 3-4	5	18.52
		L1	5	18.52
		V1	0	
		>sm1	13	48.15
		R1 lat	2	7.41
		R1 basal	4	14.81
